# Evaluation of Educational Feedback in Urology Training: A Survey-Based Assessment of Trainees and Program Directors

**DOI:** 10.7759/cureus.51716

**Published:** 2024-01-05

**Authors:** Kyle Waisanen, Gaganjot Parmar, Nathaniel Iskhakov, Daniel Baetzhold, Ellen Lutnick, Finn Henning, Kiana Saade, Matthew Peterson, Nader Nader, K. Kent Chevli

**Affiliations:** 1 Urology, Lee Physician Group Urology, Fort Myers, USA; 2 Urology, University at Buffalo Jacobs School of Medicine and Biomedical Sciences, Buffalo, USA; 3 VA (Veteran Affairs) WNY (Western New York) Health Care System, Buffalo VA Medical Center, Buffalo, USA

**Keywords:** surgical training, fellows, residents, education, feedback

## Abstract

Objective: Our objective was to evaluate current satisfaction with the feedback provided during post-graduate urological training, including the quality and frequency of feedback, with participants consisting of both trainees and program directors. Additionally, we aimed to identify areas for future improvement in resident and fellow-level urological training.

Methods: Graduating residents, fellows, and program directors from accredited residency/fellowship programs in the United States were surveyed. A total of 575 surveys were sent out. Information on feedback frequency, quality, form, and satisfaction was collected using applicable multiple-choice responses and a five-point Likert scale. An open-ended question gathered suggestions for improving current feedback processes. A chi-square test of independence was used to compare the responses to individual questions.

Results: Ninety-two respondents answered our survey: 22 residents, 18 fellows, 25 residency program directors (PDs), and 27 fellowship PDs. The distribution of age, race, and gender categories was not significantly different between PDs and trainees. However, there was a significant difference in their subspecialties and American Urological Association (AUA) sections. The majority of fellowship PDs, residency PDs, fellows, and residents (88 total) reported verbal feedback as the predominant method within their practice. This was followed by written (68 total), electronic (54 total), and app-based feedback (19 total).

Conclusion: Our study suggests that there may be a need for ongoing improvement or standardization of feedback mechanisms in the field of urological training and highlights the perceived discrepancies between learners and educators.

## Introduction

The Accreditation Council for Graduate Medical Education (ACGME) describes feedback as "ongoing information provided regarding aspects of one's performance, knowledge, or understanding." Constructive and meaningful feedback is a core faculty requirement for residency training and education according to ACGME guidelines. The recommendations emphasize frequent feedback; however, the council does not provide a medium for required formal documentation of the provided feedback. There is also an expectation for residents to provide self-directed feedback by reflecting on their own work and production. When used appropriately, feedback becomes an important tool to strengthen leadership qualities and behaviors, which are crucial for the development of practicing surgeons [1]. A lack of feedback has been shown to limit improvement as repetition of a procedure alone cannot assure development of skills. In previous studies, residents often reported a need for improved feedback, implying the delivery method of feedback is flawed or perceptions of feedback differ between program faculty and trainees [2]. It is important to explore the different types of feedback mechanisms and their individual benefits, barriers to feedback, and discrepancies between trainees and teachers on perceptions of feedback.

Resident evaluation takes many forms during their training and can help shape a surgical resident's experience before, during, and after a surgical case. In theory, attendings can provide feedback verbally, in writing, or digitally. This interaction can be presented in both formal and informal contexts, with both options having the potential for meaningful education and interaction for both educators and learners. In one interview-based study, residents reported their formal feedback comes in written form, consisting of monthly evaluations and semiannual reviews. Some of these residents stated this information was far from optimal, as they received it long after the surgical experience, and the comments were often too vague to be useful. This study also reported "on the whole, residents agreed that in-person feedback was rare." Another variety of informal feedback consisted of subjective, subtle processes like reading team members' facial expressions or body language and paying attention to team dynamics and interpersonal interactions [1]. Concern over the frequency of informal feedback has been echoed in other studies, which found that residents report receiving less feedback than attending physicians report giving [3]. Oral feedback appears to dominate the learning context for surgical residents. One systematic review, which included three interventional studies, reported that all three studies utilized only oral feedback mechanisms [4].

While a great deal of educational feedback in the day-to-day setting of surgical residency occurs in an informal setting, the value of structured feedback models cannot be overlooked. One literature review identified three methods for structured feedback sessions: the Non-Technical Skills for Surgeons in Denmark (NOTSSdk) form, the SHARP five-step feedback tool (Set learning objectives, How did it go?, Address concerns, Review learning points, and Plan ahead), and the BID (Briefing, Intra-operative, Debriefing) teaching model [3]. A fourth study utilized a 60-minute feedback session with an expert surgeon reviewing a video of a resident performing surgery. Similarly, Trehan et al. reported that feedback consistently improved surgical performance, especially with the use of a surgical video review with an expert as the intervention to provide residents with feedback. McKendy et al. reported, "not only does a structured feedback model lead to better feedback, it can lead to tangible gains in operative skill."

Barriers to feedback can be classified as structural or personal. Structural barriers include providing feedback in the setting of an operating room, a busy clinic, or a shared space with multiple providers, or there may even be a lack of required physical space to give private feedback. Some other reasons may include a lack of time or conflicting priorities. Personal barriers to feedback include the culture of the residency program, the personal or professional relationship one has with the individual receiving feedback, and difficulty discussing sensitive or negative evaluation topics. Some individuals may have an inability to present sensitive subjects in a constructive manner. Given this discomfort and the transient nature of residency teams, some residents avoided giving feedback altogether [1]. Another barrier to feedback that has been identified in recent years is the gender of the recipient or educator. In a study assessing differences in experience receiving feedback between men and women, residents reported significant differences. Women encountered conflicting feedback from supervisors regarding confidence and assertiveness (e.g., sometimes told to be more assertive, other times to be less), often resulting in self-censorship; similar feedback was rarely noted by men [5]. Power differentials may constrain feedback from more junior educators to senior residents and faculty and there may be a fear of retaliation. Residents’ main concerns with providing upward feedback to attending physicians are concerns of adverse consequences, beliefs that supervisors will neither accept feedback nor change their behaviors, and avoidance of constructive upward feedback [6].

It is apparent that there exists a discontinuity between the perceptions of feedback among current residents and program directors. Considering the spectrum of ways in which feedback is provided and its significance in the surgical field, it is essential to explore additional avenues to improve. For these reasons, we aimed to determine and compare the different methods by which urology trainees are assessed, the perceived frequency of feedback, overall satisfaction, and recommendations for improvement.

## Materials and methods

Program directors, graduating urology residents, and subspecialty fellows from accredited urology residency and fellowship programs in the United States took part in a cross-sectional, anonymous survey. The survey was designed to gather information on the different methodologies of feedback that participants received during their training, the frequency of feedback, the quality of feedback, their satisfaction with the feedback given, and suggestions for improvements to the feedback process. The Institutional Review Board at the State University of New York at Buffalo reviewed and approved the study, which was deemed exempt due to minimal risk to participants.

The authors developed a fourteen-question survey and pilot-tested it with residents from our program to ensure clarity before it was distributed. The survey was hosted online via the Survey Monkey platform and disseminated via a mass email to the Program Directors’/Coordinators’ listserv. A total of 575 surveys were sent. The email contained information about the study and a link to the Survey Monkey web-based application. Participants received no form of compensation for participating in this study. This survey was open to completion on Survey Monkey for six months, from January 2021 to July 2021. A copy of the survey can be found in the Appendix.

The survey used in this study consists of an initial demographic questionnaire. The demographics questionnaire first asked the participants to categorize their age, gender, followed by their post-fellowship plans, and a questionnaire including the frequency, method, and their overall satisfaction with the feedback received. Lastly, the participants were allowed to provide their recommendations for improvement in an open-ended question format. These surveys were distributed to graduating residents/fellows and residency/fellowship program directors.

Results and responses were collected using the Survey Monkey software and analyzed with a chi-square test of independence to determine significance. Information on feedback frequency, quality, form, and satisfaction was collected using applicable multiple-choice responses and a five-point Likert scale. An open-ended question gathered suggestions for improving current feedback processes. All statistical analyses were performed using the IBM SPSS Statistics for Macintosh, Version 28 (Released 2021; IBM Corp., Armonk, New York). Calculations included descriptive analyses of the responses. The frequency of responses was quantified for each multiple-choice question. Data were graphed to better present the information. A chi-square test was used to compare the responses to individual questions. A P-value of 0.05 was considered statistically significant.

## Results

Methods of feedback

Ninety-two respondents answered our survey; 22 residents, 18 fellows, 25 residency PDs, and 27 fellowship PDs. A response rate of 16% for all trainees and PDs was noted. The descriptive statistics of the respondents are shown in Table 1. The distribution of age, race, and gender categories was not significantly different between PDs and trainees. However, there was a significant difference in their subspecialties and American Urological Association (AUA) sections. The majority of fellowship PDs, residency PDs, fellows, and residents (88 total) reported verbal feedback as the predominant method for feedback within their practice. This was followed by written (68 total), electronic (54 total), and app-based feedback (19 total).

**Table 1:** Descriptive Statistics (N=Total Respondents)

	Respondents (n)	P-value
Age (N=41)		0.585
20-30	4	
30-35	27	
35-40	9	
40-45	1	
Race (N=41)		0.833
White or Caucasian	28	
Black or African American	0	
Hispanic or Latino	4	
Asian or Asian American	5	
Native Hawaiian or other Pacific Islander	0	
Another race	1	
Prefer not to answer	3	
Gender (N=41)		0.194
Male	30	
Female	9	
Non-binary	1	
Prefer not to answer	1	
AUA Section (N=95)		0.194
South Central	9	
North Central	21	
Southeastern	12	
Mid-Atlantic	5	
New York	7	
Northeastern	12	
New England	11	
Not affiliated with AUA section	6	
Subspecialty (N=48)		0.023
Female Pelvic Medicine and Reconstruction	3	
Pediatrics	6	
Oncology	11	
Endourology	18	
Infertility	2	
Male Reconstruction	6	
Fertility	1	
Voiding Dysfunction	1	

Frequency of feedback

**Figure 1:**
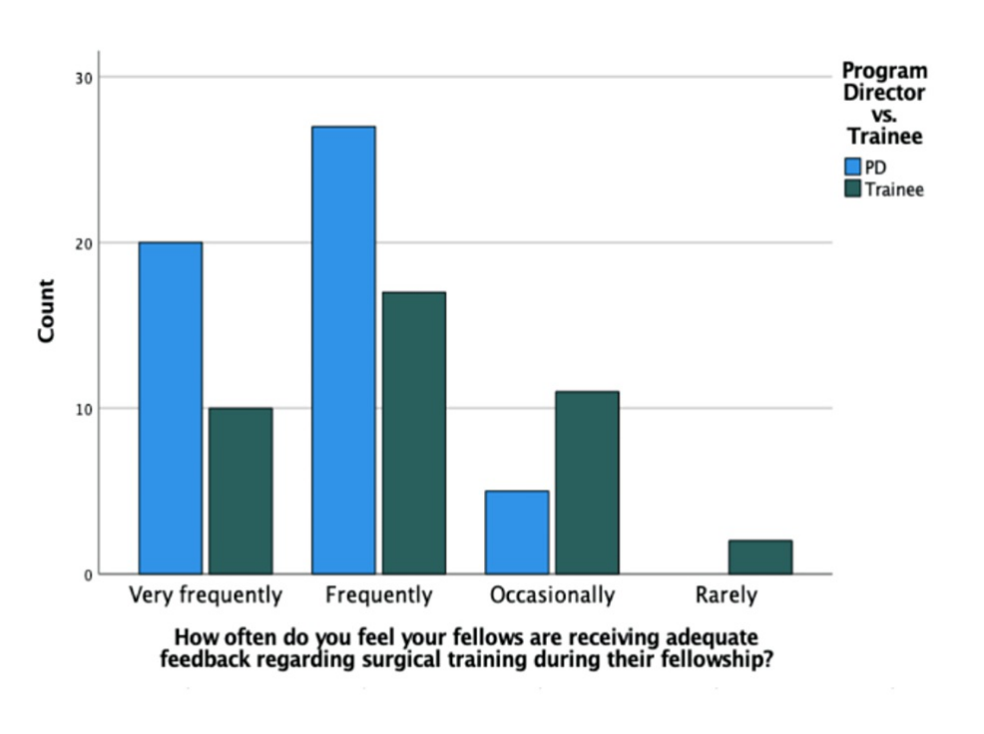
Perceptions regarding feedback on surgical training

**Figure 2:**
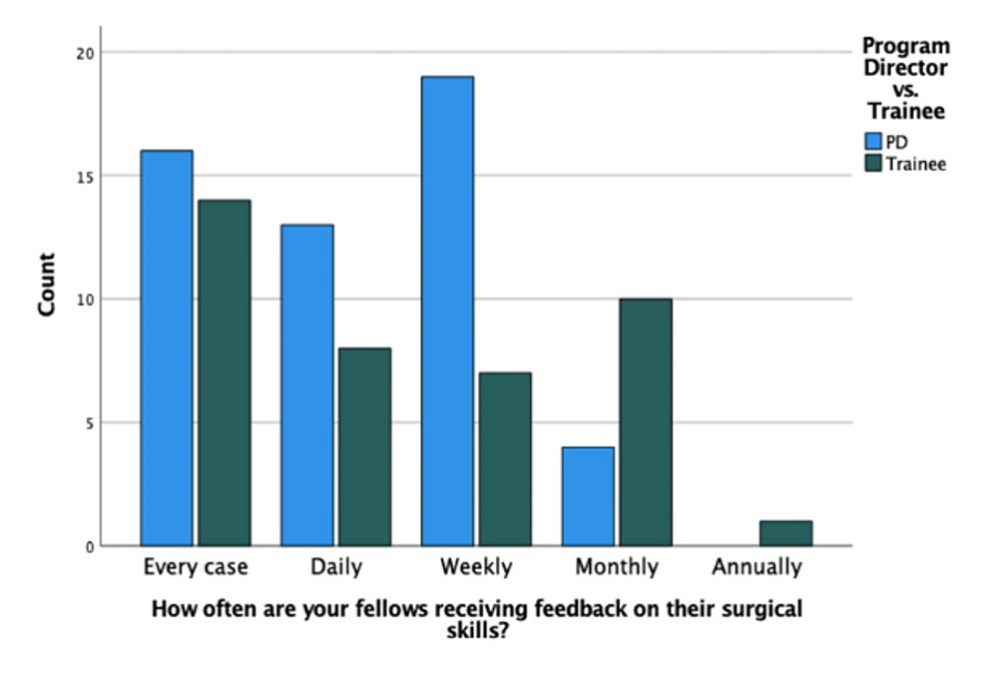
Perceptions regarding feedback on surgical skills

There was a significant difference in the perceived frequency of feedback regarding surgical training between PDs and trainees. Figure 1 shows more PDs (20/52; 38%) than trainees (10/40; 25%) reported that trainees received adequate feedback "very frequently." Similarly, 5/52 (9.6%) PDs and 11/40 (28%) trainees reported that trainees received adequate feedback "occasionally." Out of all respondents, only two residents (5% of total trainees) reported receiving adequate feedback "rarely." A plurality of participants reported that trainees received feedback on every surgical case (30/92), although there was no significant difference between the groups (P=0.123). In Figure 2, the second most popular response was that trainees received feedback on a weekly basis; 84% of participants reported that trainees receive feedback on their surgical skills at least on a weekly basis (77/92). Only one fellow reported that they receive surgical feedback annually.

Feedback satisfaction

**Figure 3:**
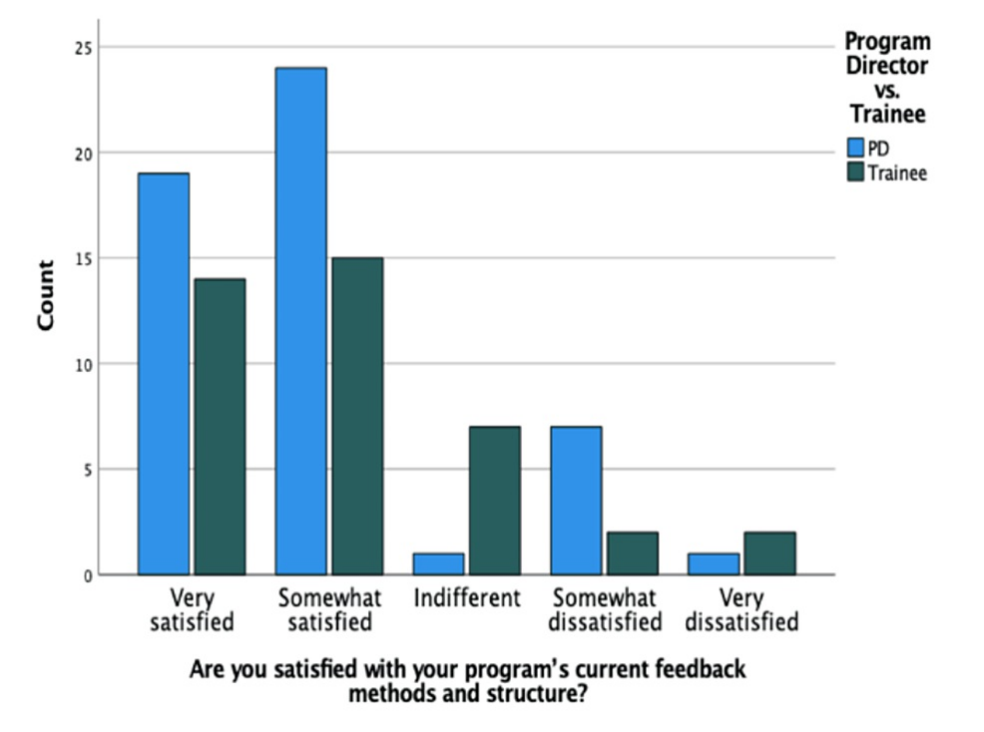
Perceptions of satisfaction with the current feedback model

There was not a significant difference in the overall feedback satisfaction between PDs and trainees. Figure 3 shows that a predominance of PDs (22/27, 81.5%) reported a satisfaction level of somewhat satisfied or greater with their program’s current feedback methods and structures. Furthermore, 29/40 (72.5%) trainees reported a satisfaction level of somewhat satisfied or greater. In total, 12/92 (13%) of all participants reported dissatisfaction with the feedback received. Ten respondents noted that common suggestions for improving feedback from PDs included increasing standardized structure and utilizing electronic applications. Trainees suggested more structured feedback and providing real-time instruction and feedback delivery in clinical settings without compromising autonomy. In terms of open-ended questions, when it came to improving current resident education and/or feedback, 32% of the respondents chose not to answer, while 21% suggested some sort of change in feedback. Seven of the 91 participants suggested more surgical time, followed by four and five participants aiming for introducing apps and increasing autonomy, respectively. And 82% of the total participants chose not to comment on how to help improve insight into overall resident preparedness for performing general urologic procedures after graduation. Of those who answered, increasing autonomy and surgical exposure were the most common responses.

## Discussion

Our study addresses an essential aspect of residency training through trainee feedback mechanisms and the perceived dynamics of both learners and educators on training quality. Our study’s findings emphasize the need for further discussion and improvement of current feedback mechanisms for urology-specific graduate-level training. Recognizing the perceived discrepancies between learners and educators has the potential to enhance the quality of urology resident and fellow education through the promotion of more effective feedback practices during essential training years. In post-graduate medical and surgical training, constructive and meaningful feedback is essential to learner advancement through recognition and intervention for both trainees' areas of strength and areas requiring improvement. By exploring the existing guidelines and highlighting the discrepancy between trainees and program directors' perception of feedback, our study aimed to assess the overall quality of feedback in post-graduate training programs to aid further improvements in the feedback provided during training. Addressing this disparity may lead to improved communication and understanding between program directors and trainees, ultimately adding to the confidence of graduating trainees and benefiting the overall urological training experience.

Additionally, feedback plays a crucial role in the development of surgical skills and leadership qualities. By investigating the delivery methods and perceptions of feedback, this research helps to identify potential flaws or gaps in the current feedback process. The findings can help program faculty and trainees understand the importance of effective feedback and work towards improving the delivery and reception of feedback in surgical training. A noteworthy finding is the prevalence of participants reporting that trainees receive feedback on a weekly basis, and a significant number of participants, both PDs and trainees, reported that feedback is provided on every surgical case. This consistency indicates that there is a shared understanding of the necessity of regular feedback in surgical training, regardless of the role of the respondent, and that both parties understand that feedback is an integral part of each surgical procedure, reflecting a commitment to trainee development. The perceived significant value of feedback is supported by prior studies citing that residents lay emphasis on the necessity for persistent implementation of feedback [1] and that PDs indicate program success is supported by feedback that is frequent and actionable [2].

The most important implication of this study is perhaps the simplest: our study found a notable gap in feedback satisfaction exists between urology trainees and program directors for their current training practices. More PDs reported that trainees received adequate feedback "very frequently" compared to trainees themselves. This suggests that PDs might have a more optimistic view of the feedback process than trainees. Our finding is in accordance with previous studies showing significant differences between resident and faculty perceptions of feedback. Faculty members believed they provided consistent and adequate feedback post-surgery, but residents did not have the same understanding [7]. This gap pervades the topic, including both frequency and methods of feedback, highlighting the need for more open and trainee-directed communication between trainees and program directors.

Furthermore, our study indicates a need for assessing feedback itself, allowing trainees to express what they need to improve their training and, more importantly, how they may best receive this information in a meaningful way. Other studies performed within numerous different specialties have noted similar outcomes. The delivery method of feedback plays a role in feedback satisfaction. A study performed amongst neurosurgery residents indicated that neurosurgery residents found face-to-face feedback to be the most useful while other methods such as summative or “end of the quarter” feedback were not helpful [8]. Our data showed a majority of participants were satisfied with the feedback within their program, however, 13% of all participants reported dissatisfaction with the feedback received. The data gathered also raises questions about the standardization of feedback. Would guidelines for certain types of feedback at certain intervals help trainees find common ground, and would such guidelines improve feedback satisfaction? Would such interventions lead to improvements in patient care? Or perhaps the solution would be to tailor feedback to the diverse preferences and expectations of trainees and to continue to improve feedback practices. Future studies will be critical in answering these questions and more in order to help build consensus between trainees and program directors throughout the field of urology.

Recent studies have also shown that there seems to be an incongruence between the resident and program director’s (PD’s) perception regarding feedback in the immediate post-operative period. While PDs believe they are providing adequate feedback, current residents in multiple sub-specialties disagree and believe they are not being properly evaluated. Such differences in perceptions lead to a negative work environment causing frustration among both parties [7]. In the past, trainees were expected to learn from repeated exposure. However, the traditional concept of “see one, do one, teach one” cannot be applied to the current residents, as the time spent by the current residents in procedure-based training has decreased. Studies have found that residents of all specialties spend only 6% to 21% of their total residency hours in the operating room [9]. Moreover, the currently training millennial generation leans towards training with technology through constant exposure in an environment of rapid technological advancements. Therefore, it is important to explore new ways to cater to the current, technologically advanced team of trainees.

Studies have further shown that the current generation of residents is more likely to find real-time feedback more effective, including comparing a trainee’s performance to a predetermined standard. Videotape learning for practicing should be utilized especially in the age where minimally invasive endoscopic approaches are becoming more dominant [10]. Additionally, with the rising accessibility of smartphones, digital feedback in the form of software applications (apps) has been broadly implemented in surgical training programs. These apps include O-SCORE, Practice XYZ, “Zwisch me!!”, SIMPL, the UCLA Peds Surgery app, and more [11]. In particular, the O-SCORE app was well received in one residency program it was applied to, with 81% of residents reporting that they had a positive impact on their training and 66% of faculty feeling that the application had a positive effect on the quality of feedback they provided [12]. Karim et al. (2017) reported that implementation of the “Zwisch me!!” app led to residents receiving identifiable feedback in a high proportion of cases, and over half of those comments were specific enough to lead to trainee improvement [13]. Initial studies are optimistic that apps can improve both the efficacy and efficiency of feedback in surgical training, but further investigation is warranted to elucidate the details of how this shift in medium will affect the field on a broader level [14,15].

While this study offers a useful snapshot of the current state of feedback found in a small sample of urological training today, the survey needs to be updated and improved to be utilized in future data collection. One limitation of our study is the small sample size; therefore, an increase in sample size may help increase generalizability. This may be accomplished through any of the following methods: regular accrual by the AUA survey, linking to the inservice training, or applied through faculty-guided training education. Allowing more time for data collection and sending “reminder” emails to potential participants would both likely increase participation in the survey. Another way to improve the study would be to further differentiate trainees based on their experience as physicians. Surveying trainees as they progress from interns to residents and to fellows could provide more insight into how the stages of urological training demonstrate gaps or improvement along the training continuum between trainees and attendings. This study may also be expanded along with the evolution of feedback delivery methods as electronic and application-based feedback has become more prevalent. Feedback methods, both technological and otherwise, will undoubtedly change in the coming years, and this study hopes to lay the groundwork to help track these shifts, utilizing new data as it becomes available.

Future iterations of this survey have the potential to inform on more than satisfaction and preferences; they can be used to direct real-world changes in surgical training. One way to achieve this would be to open up the data collection to more trainee and program director ideas. Participants could identify barriers to improvements to existing feedback frameworks, and these data could be used to overcome obstacles that could not be otherwise commented on in an academic survey.

## Conclusions

Despite formal documentation requirements through the ACGME, verbal feedback remains the most common delivery method for providing feedback to urology trainees. PDs reported delivering feedback at an adequate frequency, whereas trainees reported not receiving feedback frequently enough. Our study suggests that there may be a need for ongoing improvement or standardization of feedback mechanisms in the field of urological training. It also highlights the differences of opinion between learners and educators regarding the feedback that trainees receive. This questionnaire could also serve as a marker for ongoing improvements in the field of urological training. Used on a broad scale like this study or implemented within one training program, this survey can provide useful insight into the quality of training and even gauge the success of new interventions to improve feedback for trainees.

## Appendices

**Table 2:** The Survey Form Used in This Study

Fellowship Directors
1. Basic demographics
a. AUA section
b. Length of time in fellowship director position
c. Fellows per year
d. Length of fellowship
e. What is your fellowship program’s urologic subspecialty?
i. Urologic Oncology
ii. Urologic Reconstruction
iii. Endourology
iv. Men’s Health/Andrology/Infertility
v. FPMRS
vi. Voiding Dysfunction
vii. Pediatric Urology
viii. MIS
ix. Other
2. How would you rate the average preparedness of your incoming fellows to perform at the level of attending a general urology practice?
a. 1–10 (1 = not prepared, 10 = very prepared)
3. How would you rate the average preparedness of your incoming fellows to enter into fellowship?
a. 1–10 (1 = not prepared, 10 = very prepared)
4. How would you rate your incoming fellows’ overall technical skills for the following categories?
a. Open surgical skills
b. Laparoscopic surgical skills
c. Robotic surgical skills
d. Endoscopic surgical skills
5. Please indicate how strongly you agree/disagree with the following statements:
a. I feel as though this program’s average incoming fellow chose to participate in a fellowship (Please answer each one)
i. To increase marketability or job availability.
ii. To better prepare themselves for academic practice.
iii. Because they do not feel independent practice-ready in regard to surgical skills.
iv. Because they do not feel independent practice-ready in regard to knowledge of urologic pathology/treatment/core curriculum.
(0–5; 0 = unable to assess, 1 = strongly disagree, 2 = disagree somewhat, 3 = neither agree nor disagree, 4 = somewhat agree, 5 = strongly agree)
6. If you have been a fellowship director for 10 years or greater, how do you feel today’s incoming fellows’ ability to perform within a general urologic practice compared to fellows from 10+ years ago?
a. More prepared
i. If you answered more prepared, why?
b. Similarly prepared
c. Less prepared
vi. If you answered less prepared, why?
7. Do you feel COVID-19 had an impact on your graduating residents’ overall comfort level of performing general urologic procedures after graduation?
a. Yes
b. No
i. If yes: What part of their training do you think most impacted
8. Do you feel COVID-19 had an impact on your graduating residents’ decision between fellowship or entering practice immediately after graduation?
a. If yes: how did it impact their decisions
9. What methods for feedback are currently in practice within your fellowship program? (Choose all that apply)
i. Paper
ii. In person
iii. Electronic
iv. App-based
v. Other
10. Do you feel your current fellows are receiving adequate feedback regarding their surgical training during your residency?
a. Always
b. Sometimes
c. Neither
d. Rarely
e. Never
11. How often do your fellows receive feedback on their surgical skills?
a. Every case
b. Daily
c. Weekly
d. Monthly
e. Annually
f. Never
g. Other
12. Are you satisfied with your program’s current feedback methods and structure?
a. Very satisfied
b. Somewhat satisfied
c. Indifferent
d. Somewhat dissatisfied
e. Very dissatisfied
i. Why are you not very satisfied and what can be improved?
13. What overall changes do you think would most improve the current resident education and/or feedback processes?
a. Open-ended question
14. Please feel free to provide any other information or comments you feel may help provide insight into your graduating residents’ overall preparedness upon graduation.
Graduating Fellows
1. Basic demographics
a. Age
b. Race
c. Sex
d. Relationship status
e. AUA section
2. What are your plans after completing the fellowship?
a. Private practice
b. Academic practice
c. Dedicated research
d. Further training
e. Undecided
3. Have your post-fellowship plans changed from when you started fellowship
a. Yes
i. If Yes, what was your original plans
1. Private practice
2. Academic practice
3. Dedicated Research
4. Further Training
5. Undecided
b. No
4. How would you rate your feeling of preparedness on entering into your fellowship-trained field?
a. 1–10
5. Will performing general urology procedures be part of your post-fellowship career?
a. If yes: please answer question 5
6. Please rate your comfort level performing the following procedures without supervision at the time of completing the fellowship from 1 to 10
a. TURP
b. Ureteroscopy
c. Radical Nephrectomy
i. Robotic
ii. Lap/Hand Assist
iii. Open
d. Partial Nephrectomy
e. PCNL
f. Radical Cystectomy
i. Robotic
ii. Open
g. Ureteral Reimplant
i. Robotic
ii. Open
h. Ileal Conduit
i. Orthotopic Neobladder
j. Hypospadias repair
k. Circumcision
l. Hydrocelectomy
m. Orchiectomy
n. Vasectomy
o. Urethral Sling
p. Penile Prosthesis
q. Free text: What would you say is your least comfortable procedure at this time
7. Will you plan on performing robotic surgical procedures after completing the fellowship?
a. If yes: Rate your comfort level of performing robot procedures without supervision 1–10
1. After discussions with faculty, do you feel you are more trained, equally trained, or less trained when compared to previous graduating residents over the past 5–10 years?
a. More trained
b. Equally trained
c. Less trained
2. Do you feel COVID-19 had an impact on your overall comfort level of performing general urologic procedures after graduation?
a. Yes
b. No
i. If yes: What part of your training do you think most impacted
8. Do you feel COVID-19 had an impact on your comfort level operating in your fellowship-trained specialty on completion of fellowship?
a. Yes
b. No
i. If yes: What part of your training do you think it most impacted
9. Do you feel COVID-19 had an impact on your decision between fellowship or entering practice immediately after graduation?
a. Yes
b. No
i. If yes: How did it impact your decision to enter practice
10. How often do you feel you are receiving adequate feedback regarding your surgical training during your fellowship?
a. Always
b. Sometimes
c. Neither
d. Rarely
e. Never
11. How often are you receiving feedback on your surgical skills?
a. Every case
b. Daily
c. Weekly
d. Monthly
e. Annually
f. Never
g. Other
12. What methods for feedback are currently in use with your fellowship program? (Choose all that apply)
i. Paper
ii. In person
iii. Electronic
iv. App based
v. Other
13. Are you satisfied with your program’s current feedback methods and structure?
a. Very satisfied
b. Somewhat satisfied
c. Indifferent
d. Somewhat dissatisfied
e. Very dissatisfied
i. Why are you not very satisfied and what can be improved?
ii. What do you think would most improve the current resident/fellowship education and feedback processes?
a. Open-ended question
14. Please feel free to provide any other information or comments you feel may help provide insight into your overall preparedness for performing urologic procedures upon graduation.
Graduating Residents
1. Basic demographics
a. Age
b. Race
c. Sex
d. Relationship status
e. AUA section
2. What are your plans after completing residency?
a. Private practice
b. Academic practice
c. Dedicated research
d. Fellowship
e. Undecided
3. Have your post-residency plans changed from when you first entered residency?
a. Yes
i. If Yes, what was your original plans
1. Private practice
2. Academic practice
3. Dedicated research
4. Fellowship
5. Undecided
b. No
4. How would you rate your feeling of preparedness on entering into a general urology setting?
a. 1–10
5. If you are going into fellowship, how would you rate your feeling of preparedness for entering into a fellowship?
a. 1–10
6. Please rate your comfort level performing the following procedures at the time of graduation without supervision from 1 to 10.
a. TURP
b. Ureteroscopy
c. Radical Nephrectomy
i. Robotic
ii. Lap/Hand Assist
iii. Open
d. Partial Nephrectomy
e. PCNL
f. Radical Cystectomy
i. Robotic
ii. Open
g. Ureteral Reimplant
i. Robotic
ii. Open
h. Ileal Conduit
i. Orthotopic Neobladder
j. Hypospadias repair
k. Circumcision
l. Hydrocelectomy
m. Orchiectomy
n. Vasectomy
o. Urethral Sling
p. Penile prosthesis
q. Free text: What would you say is your least comfortable procedure at this time
7. Is there a field in urology you feel most comfortable operating at time of graduation without fellowship training?
a. Endourology
b. Fertility
c. Oncology
d. Female Pelvic
e. Voiding Dysfunction
f. Pediatrics
g. Sexual Medicine
h. Male Reconstructive
i. No field of increased comfort
8. Is there a field in urology you feel least comfortable operating at time of graduation?
a. Endourology
b. Fertility
c. Oncology
d. Female Pelvic
e. Voiding Dysfunction
f. Pediatrics
g. Sexual Medicine
h. Male Reconstructive
i. No field of decreased comfort
9. Do you plan on performing robotic surgical procedures post-graduation?
a. If yes: Rate your current comfort level of performing robot procedures without supervision 1–10
10. After discussions with faculty do you feel you are more trained, equally trained, or less trained when compared to previous graduating residents over the past 5–10 years?
a. More trained
b. Equally trained
c. Less trained
11. Do you feel COVID-19 had an impact on your overall comfort level of performing general urologic procedures after graduation?
a. Yes
b. No
i. If yes: What part of your training do you think it most impacted
12. Do you feel COVID-19 had an impact on your decision between fellowship or entering practice immediately after graduation?
a. If yes: how did it impact your decisions
13. What was your most important educational resource during residency?
a. AUA core curriculum
b. Campbells
c. Weiders
d. Other: please list
14. What methods for feedback are currently in practice within your residency program? (Choose all that apply)
i. Paper
ii. In person
iii. Electronic
iv. App-based
v. other
15. How often do you feel you are receiving adequate feedback regarding your surgical training during your residency?
a. Always
b. Sometimes
c. Neither
d. Rarely
e. Never
16. How often are you receiving feedback on your surgical skills?
a. Every case
b. Daily
c. Weekly
d. Monthly
e. Annually
f. Never
g. Other
17. Are you satisfied with your program’s current feedback methods and structure?
a. Very satisfied
b. Somewhat satisfied
c. Indifferent
d. Somewhat dissatisfied
e. Very dissatisfied
i. Why are you not very satisfied and what can be improved?
18. What do you think would most improve the current resident education and/or feedback processes?
a. Open-ended question
19. Please feel free to provide any other information or comments you feel may help provide insight into your overall preparedness for performing general urology procedures upon graduation.
Residency Directors
1. Basic demographics
a. AUA section
b. Length of time in director position
c. Residents per year
d. Length of residency
2. How would you rate the average preparedness of your current chief residents to perform at the level of attending in a general urology practice on graduation?
a. 1–10 (1 = not prepared, 10 = very prepared)
3. How would you rate the average preparedness of your graduating residents entering into fellowship to perform general urology procedures?
a. 1–10 (1 = not prepared, 10 = very prepared)
4. How would you rate your current graduating residents' comfort levels for performing the following procedures without supervision at the time of graduation?
a. TURP
b. Ureteroscopy
c. Radical Nephrectomy
i. Robotic
ii. Lap/Hand Assist
iii. Open
d. Partial Nephrectomy
e. PCNL
f. Radical Cystectomy
i. Robotic
ii. Open
g. Ureteral Reimplant
i. Robotic
ii. Open
h. Ileal Conduit
i. Orthotopic Neobladder
j. Hypospadias repair
k. Circumcision
l. Hydrocelectomy
m. Orchiectomy
n. Vasectomy
o. Urethral Sling
p. Penile prosthesis
5. How would you rate your current graduating residents’ overall technical skills for the following categories?
a. Open surgical skills
b. Laparoscopic surgical skills
c. Robotic surgical skills
d. Endoscopic surgical skills
6. What percentage of your current graduating chief residents have elected to enter into a fellowship?
a. 0–25%
b. 26–50%
c. 51–75%
d. 76–100%
7. Please indicate how strongly you agree/disagree with the following statements:
a. I feel as though this program’s average graduating chief resident who chooses to perform fellowship does so… (Please answer each one)
i. To increase marketability or job availability.
ii. To better prepare themselves for academic practice.
iii. Because they do not feel independent practice-ready in regard to surgical or clinical skills.
iv. Because they do not feel independent practice-ready in regard to knowledge of urologic pathology/treatment/core curriculum.
v. Other
(0–5; 0 = unable to assess, 1 = strongly disagree, 2 = disagree somewhat, 3 = neither agree nor disagree, 4 = somewhat agree, 5 = strongly agree)
8. If you have been a program director for 10 years or greater, how do you feel today’s graduating chief residents’ ability to perform within a general urologic practice compare to chief residents from 10+ years ago?
a. More prepared
i. If you answered more prepared, why?
b. Similarly prepared
c. Less prepared
i. If you answered less prepared, why?
9. Do you feel COVID-19 had an impact on your graduating residents’ overall comfort level of performing general urologic procedures after graduation?
a. Yes
b. No
i. If yes: What part of their training do you think it most impacted
10. Do you feel COVID-19 had an impact on your graduating residents’ decision between fellowship or entering practice immediately after graduation?
a. If yes: how did it impact their decisions
11. What methods for feedback are currently in practice within your residency program? (Choose all that apply)
i. Paper
ii. In person
iii. Electronic
iv. App based
v. Other
12. Do you feel your current residents are receiving adequate feedback regarding their surgical training during your residency?
a. Always
b. Sometimes
c. Neither
d. Rarely
e. Never
13. How often do your residents receive feedback on their surgical skills?
a. Every case
b. Daily
c. Weekly
d. Monthly
e. Annually
f. Never
g. Other
14. Are you satisfied with your program’s current feedback methods and structure?
a. Very satisfied
b. Somewhat satisfied
c. Indifferent
d. Somewhat dissatisfied
e. Very dissatisfied
i. Why are you not very satisfied and what can be improved?
15. What overall changes do you think would most improve the current resident education and/or feedback processes?
a. Open-ended question
16. Please feel free to provide any other information or comments you feel may help provide insight into your graduating residents’ overall preparedness upon graduation
